# Supplementary material for: Multicentre Evaluation of the EUCAST Rapid Antimicrobial Susceptibility Testing (RAST) Extending Analysis to 16–20 Hours Reading Time
Source: Antibiotics (Basel). 2022 Oct 13;11(10):1404. doi: 10.3390/antibiotics11101404 (PMC9599042; doi:10.3390/antibiotics11101404)
Supplement: Supplementary file 1 [file antibiotics-11-01404-s001.zip › antibiotics-1956259-supplementary.pdf]

Table S1. Antibiotic discs used for EUCAST RAST according to microscopic examination results.

Gram Examination Result	Antibiotic Discs
Gram-negative rods	Piperacillin/tazobactam 30–6 μg, ceftazidime/avibactam 10–4 μg, Ceftolozane/tazobactam 30–10 μg, imipenem 10 μg, meropenem 10 μg, levofloxacin 5 μg, amikacin 30 μg, tobramycin 10 μg
Gram-positive cluster cocci	Cefoxitin 30 μg, gentamicin 10 μg, clindamycin 2 μg, clindamycin 2 μg + erythromycin 15 μg (test for inducible clindamycin resistance)
Gram-positive diplococci or short chain cocci	Ampicillin 2 μg, imipenem 10 μg, vancomycin 5 μg, linezolid 10 μg, gentamicin 30 μg

Table S2. Tentative zone diameter RAST breakpoints for Enterobacterales other than *E. coli*/*K. pneumoniae*.

Antimicrobial Agent	Disc Content (μg)	4 Hours			6 Hours			8 Hours			16–20 Hours		
		S ≥	ATU	R <	S ≥	ATU	R <	S ≥	ATU	R <	S ≥	ATU	R <
Piperacillin/tazobactam	30–6	17	13–16	13	18	14–17	14	18	14–17	14	17	15–16	15
Cefotaxime	5	15	12–14	12	18	14–17	14	18	15–17	15	16	14–15	14
Ceftazidime	10	15	12–14	12	16	14–15	14	17	14–16	14	18	15–17	15
Ceftazidime/avibactam	10–4	12	10–11	10	13	10–12	10	13	10–12	10	14	11–13	11
Ceftolozane/tazobactam	30–10	16	14–15	14	18	14–17	14	18	15–17	15	20	16–19	16
Imipenem	10	16	12–15	12	17	13–16	13	17	13–16	13	17	12–16	12
Meropenem	10	17	13–16	13	17	15–16	15	17	15–16	15	15	13–14	13
Levofloxacin	5	17	14–16	14	18	15–17	15	18	15–17	15	23	14–22	14
Amikacin	30	15	13–14	13	15	12–14	12	15	12–14	12	15	11–14	11
Tobramycin	10	14	12–13	12	15	11–14	11	15	11–14	11	14	11–13	11
